# The Dining Hall in the Thirteenth Century

**Published:** 1882-05

**Authors:** 


### THE DINING-HALL IN THE THIRTEENTH CENTURY.

Tables for meals in the thirteenth century were simply boards placed on trestles, and removed when the repast was over. On the table at the dais was silver plate, then a rare luxury, restricted to the highest classes, the articles being spoons, knives, plates and goblets. There were no forks, for only one fork had ever then been heard of as a thing to eat with, and this had been the invention of the wife of a Doge of Venice, about two hundred years previous, for which piece of refinement the public rewarded the lady by considering her as proud as Lucifer. Forks existed, both in the form of spice forks and fire forks, but no one ever thought of eating with them in England until they were introduced from Italy in the reign of James I., and for some time after that the use of them marked either a traveler or a luxurious, effeminate man. Moreover, there were no knives nor spoons provided for helping oneself from the dishes. Each person had a knife and spoon for himself, with which he helped himself at his convenience. People who were very delicate and particular wiped their knives on a piece of bread before doing so, and licked their spoons all over. When these were the practices of fastidious people, the proceedings of those who were not such may be discreetly left to imagination. The second table was left in a much more ordinary manner. In this instance the knife was iron and the spoon pewter, the plate a wooden trencher (never changed) and the drinking cup of horn. In the midst of the table stood a pewter salt-cellar, formed like a castle, and very much larger than we use them now. This salt-cellar acted as a barometer, not for weather, but for rank. Every one of the noble blood, or filling certain offices, sat above the salt. With respect to cooking, our fathers had some peculiarities. They ate many things that we never touch, such as porpoises and herons, and they used all manner of green things as vegetables. They liked their bread hot from the oven (to give cold bread, even for dinner, was a shabby proceeding), and their meat much underdone, for they thought that overdone meat stirred up anger. They mixed most incongruous things together; they loved very strong tastes, delighting in garlic and verjuice; they never appear to have paid the slightest regard to their digestion, and they were, in the most emphatic sense, not teetotalers. The dining-hall, but not the table, was decorated with flowers; and singers, often placed in a gallery at one end, were employed the whole time. A gentleman usher acted as butler, and a yeoman was always at hand to keep out strange dogs, snuff candles, and light to bed the guests who were not always in a condition to find their way up stairs without this help. The hours at this time were nine or ten o'clock for dinner (except on fast days, when it was noon), and three or four for supper. Two meals a day were thought sufficient for all men who were not invalids. The sick and women sometimes had a "rear-supper" at six o'clock or later. As to breakfast, it was a meal taken only by some persons, and then served in the bedchamber or private boudoir at convenience. Wine, with bread sopped in it, was a favorite breakfast, especially for the old. Very delicate or exceptionally temperate people took milk for breakfast; but though the middle ages present us with examples of both vegetarians and total abstainers, yet of both there were very few indeed, and they were mainly to be found among the religious order.—"*Not for Him*," *by Emily S. Holt.*

